# Ocular and orbital manifestations of granulomatosis with polyangiitis: a systematic review of published cases

**DOI:** 10.22336/rjo.2023.38

**Published:** 2023

**Authors:** Kayvon Ahmad Moin, Madeleine Marta Yeakle, Allison Margaret Parrill, Victoria Anne Garofalo, Allen Raphael Tsiyer, Daniel Bishev, Dhir Gala, Joshua Fogel, Alexander James Hatsis, Tyler Daniel Wickas, Prachi Anand, Marcelle Morcos

**Affiliations:** * American University of the Caribbean, School of Medicine, Cupecoy, Sint Maarten; ** Department of Family Medicine, University of South Carolina School of Medicine, Seneca, South Carolina, USA; *** Department of Business Management, Brooklyn College, Brooklyn, NY, USA; **** Department of Ophthalmology, Nassau University Medical Center, East Meadow, New York, USA; ***** Department of Rheumatology, Nassau University Medical Center, East Meadow, New York, USA

**Keywords:** ophthalmology, granulomatosis with polyangiitis, granulomas, c-ANCA, p-ANCA

## Abstract

**Objective:** Granulomatosis with polyangiitis (GPA) is an autoimmune disorder characterized by necrotizing granulomatous inflammation of small and medium-sized vessels. This systematic review aimed to highlight the most common ophthalmic manifestations and to uncover their associations with antineutrophil cytoplasmic antibody (ANCA) positivity and the presence of granulomas.

**Methods:** A literature search of PubMed, Web of Science, and Scopus electronic databases was performed from journal inception to March 21, 2021, for case reports and a series of ophthalmic GPAs. Cytoplasmic-ANCA (c-ANCA), perinuclear-ANCA (p-ANCA), and granulomas were analyzed against many ophthalmic signs and symptoms. 306 patients with GPA were retrospectively studied.

**Results:** Granulomas were present in 47.7% of our sample, c-ANCA in 59.2%, and p-ANCA in 10.8%. Scleritis was significantly associated with higher odds for c-ANCA positivity. Eye discharge, episcleritis, proptosis, and central nervous system (CNS) involvement were each significantly associated with lower odds for c-ANCA positivity. Orbital mass was significantly associated with lower odds for p-ANCA positivity. CNS involvement was significantly associated with higher odds for p-ANCA positivity (OR:3.08, 95% CI:1.02, 9.36, p=0.047) and orbital mass was significantly associated with lower odds for p-ANCA positivity.

**Conclusions:** We recommend that clinicians should consider ocular or orbital GPA in patients presenting with non-specific eye complaints, such as vision impairment, orbital mass, or proptosis, and obtain further assessments to determine the possible presence of granuloma, c-ANCA, or p-ANCA.

**Abbreviations: **GPA = Granulomatosis with Polyangiitis, ANCA = antineutrophil cytoplasmic antibody, c-ANCA = cytoplasmic-ANCA, p-ANCA = perinuclear-ANCA, CNS = central nervous system, AAVs = ANCA-associated vasculitides, SD = standard deviation, GU = genitourinary, ENT = ear nose and throat, OR = odds ratio, CI = confidence interval

## Introduction

GPA is a small-to-medium vessel vasculitis and represents a subgroup within the disorders known as ANCA-associated vasculitides (AAVs) [**1**]. AAVs are rare with incidence reported between 10 to 20 cases per million [**2**]. GPA is the most common of the AAVs, with an incidence of 5 to 10 cases per million, and a peak incidence at approximately 55 years of age [**2**]. GPA is characterized by necrotizing granulomatous inflammation and usually presents clinically with upper and lower respiratory tract granulomas, small-to-medium vessel vasculitis, and necrotizing crescentic glomerulonephritis [**3**]. However, ocular and orbital manifestations may arise in 16-78% of GPA patients, with up to 27% of GPA undiagnosed cases noting ophthalmic manifestations as the primary symptom during disease onset [**4**].

ANCAs are commonly found in patients with any of the three AAVs: GPA, eosinophilic granulomatosis with polyangiitis, and microscopic polyangiitis. These conditions commonly damage small and medium-sized arteries, arterioles, and venules via autoimmune dysfunction against leukocyte proteinase-3 for c-ANCA and myeloperoxidase for p-ANCA [**5**]. Although GPA is most commonly associated with c-ANCA, some studies report up to a third of patients positive for p-ANCA, and a small number of patients are negative for both [**5**]. There are different approaches to understanding the development of ANCAs; some propose associations with environmental causes or infections, supporting a cause of molecular mimicry, while others present associations with human leukocyte antigen DQ polymorphisms [**5**].

ANCA positivity can be of prognostic value for measuring GPA disease severity [**6**]. Recurrent or persistent ANCA titers are associated with a greater risk of worsened AAV disease progression and renal failure [**7**]. Patients with p-ANCA positivity generally have an overall worse prognosis and a higher risk of renal disease progression, such as ANCA-associated nephritis and end-stage renal disease [**8**,**9**].

The published research regarding ophthalmic GPA consists of retrospective reviews and cohort studies with sample sizes ranging from 17 to 113 patients [**10**-**12**]. We systematically reviewed and analyzed 306 patients from published case reports and case series to evaluate ophthalmic GPA. We compared the presence of granuloma, c-ANCA, and p-ANCA for ocular, orbital, and systemic signs and symptoms. 

## Methods

*Literature Search and Selection Criteria*

**Fig. 1:**
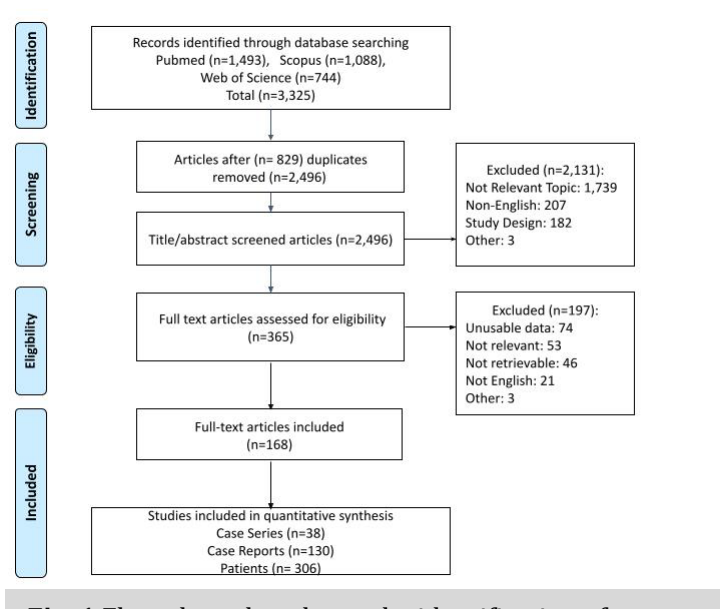
Flow chart that shows the identification of articles through the databases listed in the figure, as well as the screening, eligibility, and inclusion criteria. “n” represents the number of articles. The last box at the end of the flow chart indicates the number of studies that were included for quantitative analyses for case series, and case reports, as well as the total number of patients

A systematic search of PubMed, Web of Science, and Scopus electronic databases was performed from database inception to March 21, 2021 (**Fig. 1**). The search terms included: orbital OR ocular AND Wegener’s OR Wegener OR polyangiitis OR granulomatosis. The inclusion criteria were case reports or case series with patients diagnosed with ocular GPA written in the English language. No restrictions were made regarding age, gender, sex, race/ethnicity, or country. Exclusion criteria were literature reviews, retrospective/prospective cohorts, and experimental designs. The abstract and full-text screening was completed by two authors per article. Articles were excluded if they lacked clinical extractable data (e.g., description of symptoms, ANCA positivity, and/or treatment details). The review team of five people resolved any discrepancies.

*Variables*

Demographics were age at symptom presentation (years), sex (female/male), and previous GPA diagnosis (no/yes). Ocular signs and symptoms were eye pain, vision impairment, hyperemia/conjunctival injection, eye discharge, corneal ulcer, peripheral ulcerative keratitis, scleritis, necrotizing scleritis, episcleritis, conjunctivitis, retinitis, uveitis, pupillary defect, color vision ambiguity, optic neuritis, and ocular hypertension all reported as yes or no/unknown. Orbital signs and symptoms included proptosis, eyelid edema, xanthelasma, ptosis, diplopia, lacrimal gland involvement/dacryocystitis, orbital mass, pseudotumor, extraocular muscle involvement, oculomotor nerve involvement/palsy, orbital bone erosion, all recorded as yes or no/unknown. Systemic signs and symptoms extracted were weight loss, headache, genitourinary involvement, pulmonary involvement, central nervous system involvement, and ear, nose, and throat involvement, all recorded as yes or no/unknown. Outcomes from histology and laboratory findings were the presence of granulomas, c-ANCA positivity, and p-ANCA positivity, all recorded as yes or no/unknown. 

*Statistical Analysis*

Descriptive statistics consisted of mean and standard deviation for the continuous variables and frequency and percentage for the categorical variables. Univariate inferential analyses consisted of analysis of variance for the continuous variable of age and either the Pearson chi-square statistic or the Fisher’s exact test when the expected cell size was < 5 for the categorical variables. Any variable statistically significant in the univariate analyses was included in a multivariate logistic regression analysis. All p-values were two-tailed with alpha for significance at p<0.05. IBM SPSS Statistics version 28 was used for all analyses (**Table 1**-**4**).

**Table 1:** Whole sample description and comparisons for granuloma

Variable	Whole Sample # (%) (n=306)	No # (%) (n=160)	Yes # (%) (n=146)	p-value
*Demographics*				
Age at symptom presentation (years) [mean, SD] (n=297)	47.9 (17.8)	49.8 (17.42)	45.8 (18.04)	0.054
Sex (male)	167 (54.6)	93 (58.1)	74 (50.7)	0.19
Previous GPA diagnosis (yes)	94 (30.7)	62 (38.8)	32 (21.9)	0.001
*Ocular Signs and Symptoms (yes)*				
Eye pain	121 (39.5)	59 (36.9)	62 (42.5)	0.32
Vision impairment	172 (56.2)	89 (55.6)	83 (56.8)	0.83
Hyperemia/conjunctival injection	73 (23.9)	36 (22.5)	37 (25.3)	0.56
Eye discharge	22 (7.2)	10 (6.3)	12 (8.2)	0.51
Corneal ulcer	22 (7.2)	9 (5.6)	13 (8.9)	0.27
Peripheral ulcerative keratitis	41 (13.4)	26 (16.3)	15 (10.3)	0.13
Scleritis	73 (23.9)	37 (23.1)	36 (24.7)	0.75
Necrotizing scleritis	31 (10.1)	11 (6.9)	20 (13.7)	0.048
Episcleritis	25 (8.2)	13 (8.1)	12 (8.2)	0.98
Conjunctivitis	30 (9.8)	18 (11.3)	12 (8.2)	0.37
Retinitis	18 (5.9)	9 (5.6)	9 (6.2)	0.84
Uveitis	34 (11.1)	16 (10.0)	18 (12.3)	0.52
Pupillary defect	25 (8.2)	17 (10.6)	8 (5.5)	0.10
Color vision ambiguity	6 (2.0)	0 (0.0)	6 (4.1)	0.01
Optic neuritis	44 (14.4)	22 (13.8)	22 (15.1)	0.74
Ocular hypertension	17 (5.6)	5 (3.1)	12 (8.2)	0.052
*Orbital Signs and Symptoms (yes)*				
Proptosis	123 (40.2)	54 (33.8)	69 (47.3)	0.02
Eyelid edema	68 (22.2)	27 (16.9)	41 (28.1)	0.02
Xanthelasma	6 (2.0)	3 (1.9)	3 (2.1)	1.00
Ptosis	36 (11.8)	12 (7.5)	24 (16.4)	0.02
Diplopia	55 (18.0)	24 (15.0)	31 (21.2)	0.16
Lacrimal gland involvement/dacryocystitis	74 (24.2)	38 (23.8)	36 (24.7)	0.85
Orbital mass	132 (43.1)	45 (28.1)	87 (59.6)	<0.001
Pseudotumor	35 (11.4)	15 (9.4)	20 (13.7)	0.24
Extraocular muscle involvement	99 (32.4)	43 (26.9)	56 (38.4)	0.03
Ocular motor nerve involvement/palsy	23 (7.5)	14 (8.8)	9 (6.2)	0.39
Orbital bone erosion	38 (12.4)	17 (10.6)	21 (14.4)	0.32
*Systemic Signs and Symptoms (yes)*				
Weight loss	27 (8.8)	13 (8.1)	14 (9.6)	0.65
Headache	54 (17.6)	24 (15.0)	30 (20.5)	0.20
GU involvement	64 (20.9)	30 (18.8)	34 (23.3)	0.33
Pulmonary involvement	109 (35.6)	53 (33.1)	56 (38.4)	0.34
CNS involvement	20 (6.5)	9 (5.6)	11 (7.5)	0.50
ENT involvement	68 (22.2)	33 (20.6)	35 (24.0)	0.48
*Outcome*				
Granuloma (yes)	146 (47.7)	---	---	---
c-ANCA (positivity)	181 (59.2)	---	---	---
p-ANCA (positivity)	33 (10.8)	---	---	---

**Table 2:** Comparisons for c-ANCA positivity

Variable	No # (%)(n=125)	Yes # (%)(n=181)	p-value
*Demographics*			
Age at symptom presentation (years) [mean, SD] (n=297)	47.2 (18.27)	48.4 (17.48)	0.57
Sex (male)	74 (59.2)	93 (51.4)	0.18
Previous GPA diagnosis (yes)	31 (24.8)	63 (34.8)	0.06
*Ocular Signs and Symptoms (yes)*			
Eye pain	54 (43.2)	67 (37.0)	0.28
Vision impairment	65 (52.0)	107 (59.1)	0.22
Hyperemia/conjunctival injection	27 (21.6)	46 (25.4)	0.44
Eye discharge	15 (12.0)	7 (3.9)	0.01
Corneal ulcer	11 (8.8)	11 (6.1)	0.37
Peripheral ulcerative keratitis	8 (6.4)	33 (18.2)	0.003
Scleritis	16 (12.8)	57 (31.5)	<0.001
Necrotizing scleritis	9 (7.2)	22 (12.2)	0.16
Episcleritis	15 (12.0)	10 (5.5)	0.04
Conjunctivitis	14 (11.2)	16 (8.8)	0.50
Retinitis	3 (2.4)	15 (8.3)	0.03
Uveitis	11 (8.8)	23 (12.7)	0.29
Pupillary defect	9 (7.2)	16 (8.8)	0.61
Color vision ambiguity	2 (1.6)	4 (2.2)	1.00
Optic neuritis	18 (14.4)	26 (14.4)	0.99
Ocular hypertension	5 (4.4)	12 (6.6)	0.32
*Orbital Signs and Symptoms (yes)*			
Proptosis	63 (50.4)	60 (33.1)	0.002
Eyelid edema	34 (27.2)	34 (18.8)	0.08
Xanthelasma	3 (2.4)	3 (1.7)	0.69
Ptosis	15 (12.0)	21 (11.6)	0.92
Diplopia	29 (23.2)	26 (14.4)	0.048
Lacrimal gland involvement/dacryocystitis	35 (28.0)	39 (21.5)	0.20
Orbital mass	61 (48.8)	71 (39.2)	0.10
Pseudotumor	15 (12.0)	20 (11.0)	0.80
Extraocular muscle involvement	44 (35.2)	55 (30.4)	0.38
Ocular motor nerve involvement/palsy	12 (9.6)	11 (6.1)	0.25
Orbital bone erosion	16 (12.8)	22 (12.2)	0.87
*Systemic Signs and Symptoms (yes)*			
Weight loss	15 (12.0)	12 (6.6)	0.10
Headache	20 (16.0)	34 (18.8)	0.53
GU involvement	28 (22.4)	36 (19.9)	0.60
Pulmonary involvement	41 (32.8)	68 (37.6)	0.39
CNS involvement	14 (11.2)	6 (3.3)	0.01
ENT involvement	22 (17.6)	46 (25.4)	0.11

**Table 3:** Comparisons for p-ANCA positivity

Variable	No # (%)(n=273)	Yes # (%)(n=33)	p-value
*Demographics*			
Age at symptom presentation (years) [mean, SD] (n=297)	47.7 (17.35)	49.5 (21.49)	0.61
Sex (male)	147 (53.8)	20 (60.6)	0.46
Previous GPA diagnosis (yes)	85 (31.1)	9 (27.3)	0.65
*Ocular Signs and Symptoms (yes)*			
Eye pain	105 (38.5)	16 (48.5)	0.27
Vision impairment	154 (56.4)	18 (54.5)	0.84
Hyperemia/conjunctival injection	67 (24.5)	6 (18.2)	0.42
Eye discharge	22 (8.1)	0 (0.0)	0.15
Corneal ulcer	20 (7.3)	2 (6.1)	1.00
Peripheral ulcerative keratitis	38 (13.9)	3 (9.1)	0.59
Scleritis	65 (23.8)	8 (24.2)	0.96
Necrotizing scleritis	30 (11.0)	1 (3.0)	0.22
Episcleritis	22 (8.1)	3 (9.1)	0.74
Conjunctivitis	28 (10.3)	2 (6.1)	0.76
Retinitis	17 (6.2)	1 (3.0)	0.70
Uveitis	31 (11.4)	3 (9.1)	1.00
Pupillary defect	21 (7.7)	4 (12.1)	0.33
Color vision ambiguity	6 (2.2)	0 (0.0)	1.00
Optic neuritis	41 (15.0)	3 (9.1)	0.44
Ocular hypertension	14 (5.1)	3 (9.1)	0.41
*Orbital Signs and Symptoms (yes)*			
Proptosis	107 (39.2)	16 (48.5)	0.30
Eyelid edema	61 (22.3)	7 (21.2)	0.88
Xanthelasma	6 (2.2)	0 (0.0)	1.00
Ptosis	30 (11.0)	6 (18.2)	0.25
Diplopia	47 (17.2)	8 (24.2)	0.32
Lacrimal gland involvement/dacryocystitis	66 (24.2)	8 (24.2)	0.99
Orbital mass	125 (45.8)	7 (21.2)	0.01
Pseudotumor	29 (10.6)	6 (18.2)	0.24
Extraocular muscle involvement	87 (31.9)	12 (36.4)	0.60
Ocular motor nerve involvement/palsy	19 (7.0)	4 (12.1)	0.29
Orbital bone erosion	37 (13.6)	1 (3.0)	0.10
*Systemic Signs and Symptoms (yes)*			
Weight loss	23 (8.4)	4 (12.1)	0.51
Headache	47 (17.2)	7 (21.2)	0.57
GU involvement	59 (21.6)	5 (15.2)	0.39
Pulmonary involvement	101 (37.0)	8 (24.2)	0.15
CNS involvement	15 (5.5)	5 (15.2)	0.051
ENT involvement	65 (23.8)	3 (9.1)	0.06

**Table 4:** Multivariate logistic regression analyses for granulomas and c-ANCA positivity, and univariate analysis for p-ANCA positivity

Variable	Granuloma* OR (95% CI)	p-value	c-ANCA* OR (95% CI)	p-value	p-ANCA* OR (95% CI)	p-value
*Demographics (yes)*						
Previous GPA diagnosis	0.52 (0.30, 0.92)	0.02	---	---	---	---
*Ocular Signs and Symptoms (yes)*						
Eye discharge	---	---	0.25 (0.09, 0.66)	0.01	---	---
Peripheral ulcerative keratitis	---	---	1.84 (0.73, 4.64)	0.20	---	---
Scleritis	---	---	2.36 (1.14, 4.85)	0.02	---	---
Necrotizing scleritis	3.31 (1.43, 7.70)	0.01	---	---	---	---
Episcleritis	---	---	0.33 (0.13, 0.82)	0.02	---	---
Retinitis	---	---	3.07 (0.79, 11.92)	0.11	---	---
Color vision ambiguity	2.59x109 (<0.001, ---)	1.00	---	---	---	---
*Orbital Signs and Symptoms (yes)*						
Proptosis	0.84 (0.46, 1.53)	0.56	0.52 (0.30, 0.90)	0.02	---	---
Eyelid edema	1.50 (0.80, 2.82)	0.21	---	---	---	---
Ptosis	2.10 (0.93, 4.72)	0.07	---	---	---	---
Diplopia	---	---	0.83 (0.43, 1.60)	0.57	---	---
Orbital mass	4.00 (2.28, 7.01)	<0.001	---	---	0.31 (0.13, 0.76)	0.01
Extraocular muscle involvement	1.21 (0.68, 2.16)	0.51	---	---	---	---
*Systemic Signs and Symptoms (yes)*						
CNS involvement	---	---	0.28 (0.09, 0.82)	0.02	---	---
* Nagelkerke R Square: Granuloma=0.25, c-ANCA=0.20, p-ANCA=0.07.						

## Results

**Table 1** shows the sample characteristics. The mean age was almost 48 years, slightly more than half were male, and almost one-third had a previous GPA diagnosis. The most common ocular issues were vision impairment (56.2%) and eye pain (39.5%). The most common orbital issues were orbital mass (43.1%) and proptosis (40.2%). The most common systemic issue was pulmonary involvement (35.6%). Percentages for the outcome variables were granuloma (47.7%), c-ANCA (59.2%), and p-ANCA (10.8%).

**Table 1** shows univariate comparisons for granuloma. For demographics, previous GPA diagnoses had a significantly lower percentage for those with granuloma as compared to those without granuloma. For ocular issues, necrotizing scleritis, color vision ambiguity, and ocular hypertension each had significantly greater percentages for those with granuloma as compared to those without granuloma. For orbital issues, proptosis, eyelid edema, ptosis, orbital mass, and extraocular muscle involvement each had significantly greater percentages for those with granuloma as compared to those without granuloma. For systemic issues, none of the variables significantly differed between those with and without granuloma.

**Table 2** shows univariate comparisons for c-ANCA positivity. For demographics, none of the variables significantly differed between those with and without c-ANCA positivity. For ocular issues, peripheral ulcerative keratitis, scleritis, and retinitis each had significantly greater percentages for those with c-ANCA positivity as compared to those without c-ANCA positivity. Eye discharge and episcleritis each had significantly lower percentages for those with c-ANCA positivity as compared to those without c-ANCA positivity. For orbital issues, proptosis and diplopia each had significantly lower percentages for those with c-ANCA positivity as compared to those without c-ANCA positivity. For systemic issues, CNS involvement had a significantly lower percentage for those with c-ANCA positivity as compared to those without c-ANCA positivity.

**Table 3** shows univariate comparisons for p-ANCA positivity. None of the demographic, ocular issues or systemic issues variables significantly differed between those with and without p-ANCA positivity. For orbital issues, orbital mass had a significantly lower percentage for those with p-ANCA positivity as compared to those without p-ANCA positivity. 

**Table 4** shows multivariate logistic regression analyses for the outcome variables of granuloma and c-ANCA positivity, and univariate logistic regression analysis for p-ANCA positivity. Previous GPA diagnosis was significantly associated with lower odds for granuloma. Necrotizing scleritis and orbital mass were each significantly associated with increased odds of granuloma. In an additional multivariate analysis that included as predictors age at symptom presentation and ocular hypertension that had p-values < 0.05 in the univariate analyses, these variables were not significantly associated with granuloma, and the same significance pattern as above occurred for previous GPA diagnosis, necrotizing scleritis, and orbital mass (data not shown). Scleritis was significantly associated with higher odds for c-ANCA positivity. Eye discharge, episcleritis, proptosis, and CNS involvement were each significantly associated with lower odds for c-ANCA positivity. Orbital mass was significantly associated with lower odds for p-ANCA positivity. In an additional multivariate analysis that included the predictor of CNS involvement that had a p-value of 0.051 in the univariate analyses (data not shown), CNS involvement was significantly associated with higher odds for p-ANCA positivity (OR:3.08, 95% CI:1.02, 9.36, p=0.047) and orbital mass was significantly associated with lower odds for p-ANCA positivity.

## Discussion

We found that vision impairment (56.2%), orbital mass (43.1%), and proptosis (40.2%) had the greatest percentages for signs and symptoms of GPA. In multivariate analyses, we found that necrotizing scleritis and orbital mass were each significantly associated with increased odds for granuloma, while a previous GPA diagnosis before manifestation of ophthalmic involvement was significantly associated with lower odds for granuloma. Scleritis was significantly associated with higher odds for c-ANCA positivity, but eye discharge, episcleritis, proptosis, and CNS involvement were each significantly associated with lower odds for c-ANCA positivity. CNS involvement was significantly associated with higher odds for p-ANCA, and orbital mass was significantly associated with lower odds for p-ANCA positivity.

In our multivariate analysis for granuloma (**Table 4**), previous GPA diagnosis was significantly associated with lower odds for granuloma, while necrotizing scleritis and orbital mass were each significantly associated with increased odds for granuloma. Induction and remission maintenance pharmacological treatments of GPA aim to resolve granulomatous inflammation and vascular damage [**13**]. We suggested that the previous diagnosis of GPA was associated with lower odds for granuloma, due to successful treatment suppression of granulomatous inflammation. Also, ocular masses were associated with more severe disease course, and systemic diseases, and were found in patients with concurrent necrotizing scleritis, peripheral ulcerative keratitis, and orbital wall destruction among those with ophthalmic GPA [**12**]. We speculated that the significant association of orbital masses and necrotizing scleritis with increased odds for granuloma in our analysis may be due to their presence in the clinical course for severe GPA, and the increased inflammatory destruction led to granuloma formation. 

In our multivariate analysis of c-ANCA positivity (**Table 4**), scleritis was significantly associated with higher odds of c-ANCA positivity. We were not aware of any research on the association of scleritis with c-ANCA positivity. The sclera is an avascular structure that receives nutrients mainly from the aqueous humor [**14**]. The aqueous humor contains neutrophilic enzymes and inflammatory cytokines that reach the sclera [**15**,**16**]. We suggested that c-ANCA-positive patients may be more likely to present with scleritis due to activated neutrophil enzymes reaching the sclera via the aqueous humor. 

We found that eye discharge, episcleritis, proptosis, and CNS involvement were each significantly associated with lower odds for c-ANCA positivity. Ocular and orbital GPA is noted to involve inflammation of the lacrimal gland [**12**]. We suggested that c-ANCA positivity may be associated with higher rates of lacrimal inflammation, and therefore impairment of eye discharge. 

Proptosis secondary to GPA is due to retro-orbital granuloma formation and compression against both the optic nerve and the posterior eye [**17**]. C-ANCA may play a role in inhibiting macrophage activation via the enzymes released by nearby neutrophils, thus leading to an inhibition of granuloma formation. Previous research for CNS involvement showed that GPA patients who presented with hypertrophic pachymeningitis were more likely to be seropositive for c-ANCA and were more likely to have a worsening clinical prognosis [**18**]. This differs from our study. We suggested that neurological symptoms were typically progressive and presented later in the disease course and our data were reported from patients earlier in their disease course.

Scleritis was significantly associated with increased odds for c-ANCA positivity and episcleritis was significantly associated with decreased odds for c-ANCA positivity. The episclera is the most superficial, thin layer of the sclera that communicates with the sclera through an artery-artery anastomosis [**19**]. We proposed that the increased distance from the choroid and the decreased thickness of the episclera make it less susceptible to the c-ANCA mediated inflammation compared to that of the sclera.

In our multivariate analysis (**Table 4**), we found that orbital mass had lower odds of p-ANCA positivity. Another study found that 86% of patients with orbital mass were positive for c-ANCA, but only 25% of those with p-ANCA [**20**]. We suggested that p-ANCA-positive patients may have a less severe form of ocular and orbital GPA, and therefore have lower odds of orbital mass. 

Our data also demonstrated that CNS involvement was significantly associated with higher odds for p-ANCA. P-ANCA positivity is associated with a wide variety of AAVs – Microscopic polyangiitis, eosinophilic granulomatosis with polyangiitis, and polyarteritis nodosa. These AAVs are associated with a variety of constitutional CNS symptoms such as mononeuritis multiplex caused by damage to the vasa vasorum of the neuronal axon [**21**]. We suggested that the higher odds of CNS involvement in p-ANCA positive patients found in our study supports a growing body of evidence associating p-ANCA positivity with neuronal, and therefore CNS damage. 

One strength of this study was that this was the largest systematic review of c-ANCA positivity, p-ANCA positivity, and granulomatous formation, combining over 300 patients with ocular and/or orbital manifestations of GPA. This study had some limitations, including the exclusion of non-English language studies and observational studies. Variables such as follow-up time, time of symptom onset to diagnosis, and treatment/outcome were not included. Our reporting of “no” for variables included either not present or not mentioned in the included articles. Future studies could explore potential differences for race/ethnicity as many of our retrieved cases did not report such information. 

## Conclusion

In conclusion, ocular or orbital GPA most commonly presents with vision impairment, orbital mass, or proptosis. About half of the patients have positive c-ANCA and have granulomas on biopsy, while positive p-ANCA only occurred in approximately 10% of patients. We recommend that clinicians should consider ocular or orbital GPA in patients presenting with non-specific eye complaints such as vision impairment, orbital mass, or proptosis, and obtain further assessments to determine the possible presence of granuloma, c-ANCA, or p-ANCA. 

**Conflict of Interest statement**

The authors declare no conflict of interest.

**Acknowledgments**

We would like to acknowledge Joshua Nowitz from the American University of the Caribbean School of Medicine for his contribution to the preliminary statistical analysis of the data for this manuscript.

**Sources of Funding**

None were provided.

**Disclosures**

None.
